# Graphical and numerical diagnostic tools to assess multiple imputation models by posterior predictive checking

**DOI:** 10.1016/j.heliyon.2023.e17077

**Published:** 2023-06-13

**Authors:** Mingyang Cai, Stef van Buuren, Gerko Vink

**Affiliations:** Department of Methodology and Statistics, Utrecht University, Utrecht, the Netherlands

**Keywords:** Missing data, Multiple imputation, Model checking, Posterior predictive checking

## Abstract

**Problem:**

The congenial of the imputation model is crucial for valid statistical inferences. Hence, it is important to develop methodologies for diagnosing imputation models.

**Aim:**

We propose and evaluate a new diagnostic method based on posterior predictive checking to diagnose the congeniality of fully conditional imputation models. Our method applies to multiple imputation by chained equations, which is widely used in statistical software.

**Methods:**

The proposed method compares the observed data with their replicates generated under the corresponding posterior predictive distributions to diagnose the performance of imputation models. The method applies to various imputation models, including parametric and semi-parametric approaches and continuous and discrete incomplete variables. We studied the validity of the method through simulation and application.

**Results:**

The proposed diagnostic method based on posterior predictive checking demonstrates its validity in assessing the performance of imputation models. The method can diagnose the consistency of imputation models with the substantive model and can be applied to a broad range of research contexts.

**Conclusion:**

The diagnostic method based on posterior predictive checking provides a valuable tool for researchers who use fully conditional specification to handle missing data. By assessing the performance of imputation models, our method can help researchers improve the accuracy and reliability of their analyzes. Furthermore, our method applies to different imputation models. Hence, it is a versatile and valuable tool for researchers identifying plausible imputation models.

## 1 Introduction

Multiple imputation (MI) is a popular approach for analyzing incomplete datasets. It involves generating several plausible imputed datasets and aggregating different results into a single inference. First, missing cells are filled with synthetic data drawn from corresponding posterior predictive distributions. Next, this procedure is repeated multiple times, resulting in several imputed datasets. The parameters of scientific interest are then estimated for each imputed dataset by complete-data analysis. Finally, different estimates are pooled into one inference using Rubin's rule, which accounts for within, and across imputation uncertainty [1].

The validity of post-imputation analyzes relies on the congeniality of the imputation model and the analysis model [2], [3], [4]. If the imputation and analysis models are congenial and correctly specified, Rubin's rules will give consistent estimates [1]. Hence, a crucial part of the multiple imputation process is selecting sensible models to generate plausible values for incomplete data. However, model selection is not a trivial process in practice since there can be a wide array of candidate models to check. Therefore, researchers should consider which variables, interaction terms, and nonlinear terms are included based on the scientific interest and data features.

Despite the popularity of multiple imputation, there are only a few imputation model diagnostic methodologies. One standard diagnostic method is to compare distributions of the observed with imputed data [5], [6]. Plausible imputation models would generate imputed values with a similar distribution to the observed data. Although missing at random (MAR) mechanisms would also induce discrepancies between the observed and imputed data, any dramatic departures that the observed data features cannot explain are evidence of potential imputation model mis-specification. Reliable interpretation of the observed and imputed data's discrepancies could be derived from external knowledge about the incomplete variables and the missingness mechanisms [6].

Posterior predictive checking (PPC) has been proposed as an alternative method for the imputation model diagnostic [7], [8], [9]. PPC is a Bayesian model-checking approach that compares the replicated data drawn from the corresponding posterior predictive distribution to the observed data. If the model lacks fit, there could be a discrepancy between the replicated and observed data.

The current state-of-the-art in the imputation model diagnostic with PPC is proposed by He & Zaslavsky [8] and Nguyen et al. [9]. The inadequacies of the joint imputation model are accessed with one or more test quantities relevant to the scientific interest. To evaluate the ‘usability’ of imputation models for the test statistics, analysts compare the estimates for the complete data to their replicates. Comparisons of the complete data and its replicates ensure the calculation of test quantities with general missingness patterns.

However, the method mentioned above does not fit variable-by-variable imputation techniques. First, it focuses on evaluating the adequacy of the joint imputation model rather than evaluating individual imputation models for each variable. It may limit its ability to detect mis-specification in individual imputation models, which can be important when applying fully conditional specification [5]. Different variables require different imputation strategies. The second limitation is that relying solely on target analysis may only capture some aspects of the data relevant to the analysis. It can lead to an inaccurate model selection when the imputation model is adequate for the target statistic but not for other important aspects of the data. Finally, this method performs worse when the amount of missingness is high. The reason is that the method compares estimates of complete data to their replicates.

To address the abovementioned issues, we propose and evaluate a new implementation of posterior predictive checking for imputation techniques. The general idea is that if the imputation model is congenial to the substantive model, the expected value of the data (whether observed or missing) is in the center of corresponding predictive posterior distributions. We compare the observed data to their posterior replicates generated under the imputation model and evaluate the posterior distributions of all observed data points. This distinguishes our approach from the posterior predictive checking of imputation models by applying target analyzes. We expect that our method:

1. can be generalized to variable-by-variable imputation techniques;
2. can identify the imputation model that conforms most to the true data-generating model;
3. can be used as a model evaluation technique to identify the better substantive analysis model;
4. can perform PPC with MICE in R [10];
5. is not sensitive to the amount of missing data.

This manuscript is organized as follows. In section 2, we review the posterior predictive checking of the imputation model by applying the target analysis proposed by He & Zaslavsky [8]. Section 3 provides an overview of the MICE package and the underlying imputation algorithm: fully conditional specification (FCS). We also further point out the necessity of extending the posterior predictive checking of the imputation model so that the diagnostics would apply to the MICE algorithm. In section 4, we evaluate the performance of the proposed diagnostic approach with simulation studies. In section 5, we show the results of simulation studies. In section 6, we apply the proposed diagnostic approach to the body mass index (BMI) data of Dutch adults. Finally, in section 7, we conclude with a discussion of our findings.

## 2 Related work

### 2.1 Posterior predictive checking

Without incomplete variables, PPC compares the observed data *y* with the replicated data $y^{rep}$, which are simulated from the posterior predictive distribution, with parameter *θ*:

$$\begin{array}{c} p(y^{rep}|y)=\int p(y^{rep}|\theta )p(\theta |y)d\theta \end{array}$$

To detect the discrepancy between the model and the data, we define test quantities *T* that connect the model diagnostics to the scientific interests and estimate them for both observed and replicated data. For example, if the substantive analysis is a regression analysis, the test quantities could be the regression coefficients. Misfits of the model with respect to the data could be summarized by the posterior predictive p-value, which is the probability that the replicated data are more extreme than the observed data, for the selected test quantities *T* [11]:

$$\begin{array}{cc} p_{B}\; & =Pr(T(y^{rep},\theta )\geq T(y,\theta )|y)\\ \; & =\int \int I_{T(y^{rep},\theta )\geq T(y,\theta )}p(y^{rep}|\theta )p(\theta |y)dy^{rep}d\theta , \end{array}$$

where *I* is the indicator function. An extreme p-value (close to 0 or 1) implies suspicion of the fit of the model since the simulation variance cannot explain a consistent discrepancy between test quantities $T(y^{rep},\theta )$ and T(y,θ).

Posterior predictive checking has been widely used for model diagnostics in applied Bayesian analysis [11], and the posterior predictive distribution is usually calculated by simulation. Suppose we have *N* draws of model parameters from its posterior distribution $\theta_{j},j=1,\ldots ,N$. We then generate replicated data for every theta $\theta_{j}$. The PPC compares test quantities based on observed data with the empirical predictive distribution of test quantities. The estimated posterior predictive p-value is the proportion of these N simulations for which $T_{j}(y^{rep},\theta )>T_{j}(y,\theta )$. Apparently, PPC's application for the imputation model diagnostic is not based on the hypothesis test perspective. Hence, there is no underlying assumed distribution for the posterior predictive p-value in this case. Instead, the posterior predictive p-value indicates whether the model would provide plausible inference based on the data for the selected test quantities.

To perform multiple imputation model checking with PPC, we compare the completed data, the combination of the observed and imputed data, with its replications. Gelman et al. [7] applied graphical PPC to visualize test quantities comparisons based on completed and replicated data. He & Zaslavsky [8] and Nguyen et al. [9] developed numerical posterior predictive checks as target analyzes for the joint imputation model. He & Zaslavsky [8] proposed two kinds of discrepancies, completed data discrepancy and expected completed-data discrepancy, and the approaches to calculate corresponding posterior predictive p-values. We briefly introduce these discrepancies and p-values for the completeness of PPC for MI models.

### 2.2 Complete data discrepancy

When there are incomplete variables, the hypothetically complete data $y_{com}$ consists of the observed data $y_{obs}$ and the missing data $y_{mis}$ ($y_{com}=(y_{obs},y_{mis})$). To assess the completed-data discrepancy $T(y_{com}^{rep},\theta )-T(y_{com},\theta )$, we draw imputed values for incomplete variables $y_{mis}$ and the replication of the complete data $y_{com}^{rep}$ from their posterior predictive distribution:

$$\begin{array}{c} p(y_{com}^{rep},y_{mis}|y_{obs})=\int p(y_{com}^{rep}|\theta )p(y_{mis}|y_{obs},\theta )p(\theta |y_{obs})d\theta . \end{array}$$

To assess the model fit, we calculate the posterior predictive p-value as:

$$\begin{array}{cc} p_{B,com}\; & =Pr(T(y_{com}^{rep})\geq T(y_{com})|y_{obs})\\ \; & =\int \int I_{T(y_{com}^{rep})\geq T(y_{com})}p(y_{com}^{rep},y_{mis}|y_{obs})dy_{com}^{rep}dy_{mis} \end{array}$$

The simulation process to estimate the p-value proposed by He & Zaslavsky [8] is:

1. Simulate *N* draws of *θ* from the corresponding posterior distribution $p(\theta |y_{obs})$
2. For each $\theta_{j},j=1,\ldots ,N$, impute $y_{mis}^{j}$ from $p(y_{mis}|y_{obs},\theta_{j})$ and simulate the replicated data $y_{com}^{rep,j}$ from $p(y_{com}^{rep}|\theta_{j})$

A $p_{B,com}$, which is close to 0 or 1, implies the discrepancy between the model and the data with respect to the selected test quantities.

### 2.3 Expected complete data discrepancy

He & Zaslavsky [8] noticed that the power of completed-data discrepancy is weakened because the variance of imputed data across complete data $y_{com}$ and replicated data $y_{com}^{rep}$ increase the variance of the test quantities. Therefore, He & Zaslavsky [8] reduced the variance of completed-data discrepancy by calculating the expectation value of missing data for each model parameter draw. The modification of p-value $p_{B,ecom}$ would be:

$$\begin{array}{cc} p_{B,ecom}\; & =Pr(E[T(y_{com}^{rep})|y_{obs}^{rep},y_{obs}]\geq E[T(y_{com})|y_{obs}^{rep},y_{obs}]|y_{obs})\\ \; & =\int \int I_{E[T(y_{com}^{rep})|y_{obs}^{rep},y_{obs}]\geq E[T(y_{com})|y_{obs}^{rep},y_{obs}]}p(y_{obs}^{rep},y_{obs})dy_{obs}^{rep}, \end{array}$$

where *E* is the notation of the expected value.

Again, the nested simulation process to calculate the p-value $p_{B,ecom}$ is:

1. Simulate $N_{1}$ draws of *θ* from the corresponding posterior distribution $p(\theta |y_{obs})$
2. For each $\theta_{j},j=1,\ldots ,N_{1}$, impute $y_{mis}^{j}$ from $p(y_{mis}|y_{obs},\theta_{j})$ and simulate the replicated data $y_{com}^{rep,j}$ from $p(y_{com}^{rep}|\theta_{j})$
3. For each j-th replicate, calculate the mean discrepancy by setting $y_{mis}^{j}$ and $y_{com}^{rep,j}$ to missing and over-imputing them with the same parameters $\theta_{j}$ over $N_{2}$ draws $y_{mis}^{j,k}$ and $y_{com}^{rep,j,k},k=1,\ldots ,N_{2}$. Calculate the difference: $D_{j,k}=T(y_{obs}^{rep,j},y_{mis}^{rep,j,k})-T(y_{obs},y_{mis}^{rep,j,k})$ over $N_{2}$ draws and then average the difference for the j-th replicate: ${\overset{¯}{D}}_{j.}=\sum_{1}^{k}D_{j,k}/k$
4. Calculate $p_{B,ecom}$ as the proportion of these $N_{1}$ estimates that are positive, ${\overset{¯}{D}}_{j.}\geq 0$

He & Zaslavsky [8] evaluated whether the PPC could detect the uncongeniality of the imputation model. Nguyen et al. [9] investigated the performance of PPC in other imputation model mis-specification scenarios, such as ignoring the response variable and auxiliary variables or failing to transform skewed variables. The PPC approach proposed by He & Zaslavsky [8] is based on the joint imputation model. The imputation model for the diagnostic is a joint distribution of the observed data, and the test quantities depend on multiple variables and parameters.

## 3 MICE package

Fully conditional specification (FCS) is a popular approach for multiple imputation. It attempts to specify an imputation model for each incomplete variable $Y_{j},j=1,\ldots ,p$ conditional on all the other variables $P(Y_{j}|Y_{-j},\theta_{j})$, with parameter $\theta_{j}$. It generates imputations iteratively over all incomplete variables after an initial imputation, such as mean imputation or random draw from observed values. Let $Y_{j}^{t}=(Y_{j}^{obs},Y_{j}^{mis(t)})$ denote the observed and imputed values of variable $Y_{j}$ at iteration *t* and $Y_{-j}^{t}=(Y_{1}^{t},\ldots ,Y_{j-1}^{t},Y_{j+1}^{t-1},\ldots ,Y_{p}^{t-1})$. Given the most recent imputations of the other incomplete variables $Y_{j}^{t}$ at iteration *t*, the algorithm of generating imputations for the incomplete variable $Y_{j}$ consists of the following draws:

$$\theta_{j}^{t}∼f(\theta_{j})f(Y_{j}^{obs}|Y_{-j}^{t},\theta_{j})Y_{j}^{mis(t)}∼f(Y_{j}^{mis}|Y_{-j}^{t},\theta_{j}^{t}),$$

where $f(\theta_{j})$ is the prior distribution for the parameter of the imputation model $\theta_{j}$. The FCS is an attractive imputation approach because of its flexibility in imputation model specification. It is known under different names: chained equations stochastic relaxation, variable-by-variable imputation, switching regression, sequential regressions, ordered pseudo-Gibbs sampler, partially incompatible MCMC, and iterated univariate imputation [12].

Multivariate Imputation by Chained Equations (MICE) is the name of software for imputing incomplete multivariate data by Fully Conditional Specification. It has developed into the de facto standard for imputation in R and is increasingly being adopted in Python (e.g., statsmodels (imputer function) & miceforest). The MICE package creates functions for three components of FCS: imputation, analysis, and pooling.

**Figure 1:**
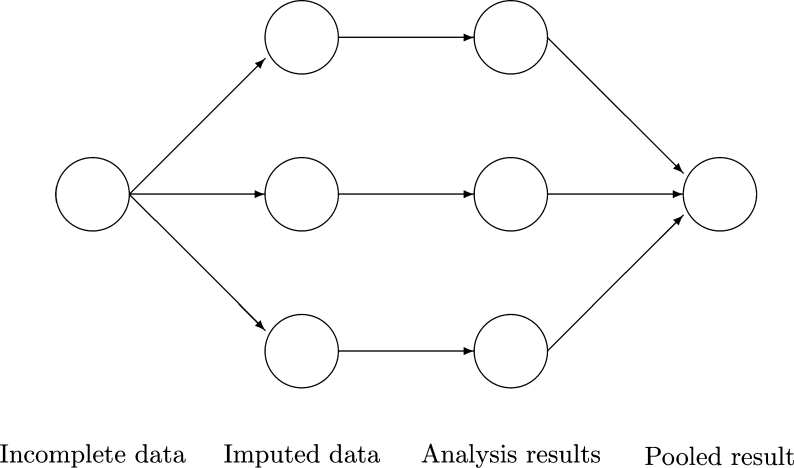
Main steps used in MICE[10].

Fig. 1 illustrates how MICE solves a missing data problem by generating 3 imputed datasets. Three imputed datasets are generated with the function **mice()**. The analysis is performed on every imputed dataset by **with()** function and combined into a single inference with the function **pool()**. The software stores the output of each step in a particular class: **mids**, **mira** and **mipo**. More details about MICE package can be found in [10].

Two features of the software motivate our research. First, the default imputation method for numerical missing data is predictive mean matching (PMM) [13].

**Figure:**
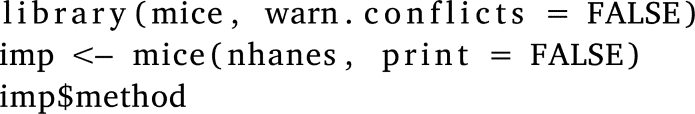

## age bmi hyp chl  ## "" "pmm" "pmm" "pmm"

It generates imputations for a missing cell from its *p* nearest points. Predictive mean matching is a semi-parametric imputation approach that is proven to perform well in a wide range of scenarios [14], [15], [16], [5], [10], [17], [18], [19], [20]. The attractive advantage of PMM is that the imputations fall consistently within the range of the observed sample space [21], [5], [17], [18], [19], [20]. For instance, PMM prevents imputing negative values for data that are strictly non-negative. Second, **mids** only stores imputed datasets, not the estimated parameters of the imputation models [22].

Based on the features of MICE package discussed above, it is necessary to investigate whether PPC could check the donor selection procedure of PMM and perform PPC based on the observed data itself instead of the target statistics. He & Zaslavsky [8] briefly discussed the approach to checking imputation models for subsets of incomplete variables. However, they assumed that the imputations of the remaining variables (excluding the incomplete variables of interest in an assessment) were adequate. Therefore, we also evaluate the performance of PPC when relaxing this assumption in the application section.

**Figure 2:**
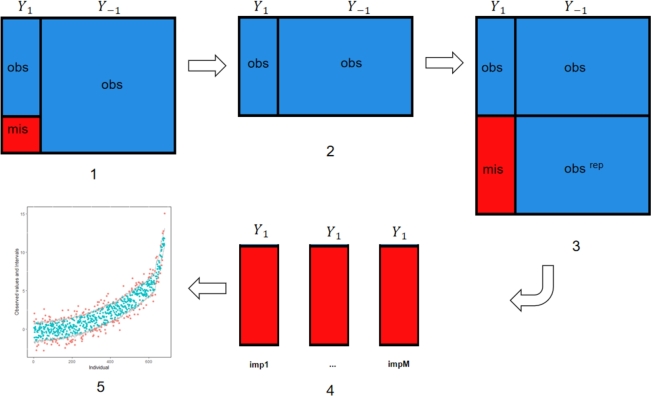
The procedure of posterior predictive checking: 1)Suppose *Y*_1_ is incomplete and all other variables *Y*_−1_ are fully observed, 2) The first step is to remove the incomplete cases from the dataset, 3) The second step is to duplicate the observed cases and remove the value of $Y_{1,obs}^{rep}$ in the duplicated part, 4) Multiply impute the missing cells $Y_{1,mis}^{rep}$ in the duplicated part, 5) Perform posterior predictive checking.

Fig. 2 shows the algorithm of the proposed diagnostic method. Implementing PPC in MICE (version 3.13.15) is straightforward. A new argument where is included in mice function, which allows us to replace the observed data by randomly drawing values from the predictive posterior distribution [23]. Here is an example of generating replications of the observed data.

**Figure:**
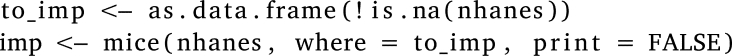

The observed data pattern may not be conducive to calculating test quantities involving multiple incomplete variables. Hence, the complete data discrepancy discussed in section 2.2 calculates test quantities based on the imputed data. In such a case, the diagnosis is sensitive to the amount of missing data. However, we propose to calculate the discrepancy based on the observed data. The proposed method compares the observed data to the corresponding predictive posterior distributions generated over multiple imputations. If the imputed model fits the observed data, the observed data will appear like a random draw from the corresponding posterior distribution.

## 4 Simulation study

We carried out a simulation study to investigate the performance of the proposed diagnostic approach. For illustrative purposes, the simulation study consisted of diagnostics under three analysis models:

1. A quadratic equation with an incomplete outcome
2. A quadratic equation with incomplete covariates
3. A generalized linear model with an incomplete binary outcome

All these scenarios are designed with several factors including missingness proportion (30%, 50%, 80%), missingness mechanisms (a missing completely at random (MCAR) and a right-tailed missing at random (MARr) mechanism), nominal levels of the confidence interval (75%, 95%) and different imputation models. We evaluated whether the proposed diagnostic method could identify the congenial imputation model for continuous and discrete incomplete variables under the first and third scenarios. We also investigated the performance of the proposed diagnostic method on the donor selection procedure of predictive mean matching under the second scenario. The sample size and the number of imputed datasets were set to be 1000 and 50 separately in all simulations.

In the simulation study, we induced missingness with the ampute() function. Generally, ampute() is a convenient function in MICE package to generate missing data for simulation purposes [24]. We considered the missing completely at random (MCAR) mechanism, where the probability of missingness is equal for every cell, as well as the right-tailed missing at random (MARr) mechanism, where higher values of covariates have a higher probability of being unobserved. In the algorithm of ampute() function, the probability of missingness is allocated with different logistic functions of the weighted sum score (*wss*), which is a linear combination of covariates correlated with the probability of missingness:

$$\begin{array}{c} wss_{i}=w_{i}x_{1i}+w_{i}x_{2i}+\ldots +w_{i}x_{mi} \end{array}$$

The weight $w_{i}$ is pre-specified to reflect the influence of the variable $x_{i}$ on the probability of missingness. For instance, if the formation of a weighted sum score is: $wss=x_{1}+x_{2}$, the probability of missingness is determined by both $x_{1}$ and $x_{2}$ with the equal effects. It is noticeable that the influence of the weights is relative. $wss=2x_{1}+2x_{2}$ will have the same effect as $wss=x_{1}+x_{2}$. More specifically, under the MARr mechanism, candidates with higher values of weighted sum score have a higher probability of being unobserved when applying the ampute() function to generate missing data.

### 4.1 Quadratic equation with an incomplete outcome

In the first simulation study, we considered a partially observed variable *Y* and a fully observed variable *X*. The data was generated from: X∼uni(−3,3), $Y=X+X^{2}+\epsilon$, where ϵ∼N(0,1). The scientific model was indeed a quadratic model. Under the MARr mechanism, the probability of missingness for the incomplete variable *Y* was completely determined by variable *X* ($wss_{Y}=X$). We considered two imputation models for the incomplete response *Y*: one is a linear regression of *Y* on *X*, and the other is a quadratic regression of *Y* on *X*. The linear regression imputation model is expected to be uncongenial and has worse performance.

### 4.2 Quadratic equation with incomplete covariates

In the second simulation study, the response variable *Y* was generated from a normal distribution: $Y=X+X^{2}+\epsilon$, where ϵ∼N(0,1) and the covariate *X* followed a standard normal distribution. In this simulation study, the response variable *Y* was completely observed, while the covariate *X* and the corresponding quadratic term $X^{2}$ were jointly missing for a fraction of the cases. There were no cases with missing cells on either *X* or $X^{2}$. Under the MARr mechanism, the probability of missingness for joint missingness of $X,X^{2}$ was completely determined by variable *Y* ($wss_{X,X^{2}}=Y$). We compared two semi-parametric methods, predictive mean matching (PMM) and polynomial combination (PC), with a parametric method, the substantive model compatible fully conditional specification (SMC-FCS) [25], [26], [27]. The PC and SMC-FCS methods are two accepted methods to impute linear regression with quadratic terms. The PC method is an extension of PMM but applies a different donor selection procedure. We expect that PC and SMC-FCS outperform PMM.

### 4.3 Generalized linear model for discrete variables

The final simulation study considered a partially observed binary *Y* and two complete covariates *X* and *Z*. The model of scientific interest was: Pr(Y=1|X,Z)=exp(X+Z)/1+exp(X+Z), where x∼uni(−3,3) and Z∼N(1,1). Under the MARr mechanism, the weights of variables *X* and *Z* in determining the probability of missingness for *Y* were set to be equal ($wss_{Y}=X+Z$). Since the logistic regression models the probability of assignment, we investigated the plot of deviance and calculated the sum of squared deviance divided by the sample size. There were two candidate models: a logistic regression of *Y* on *X* and *Z* and a logistic regression of *Y* on *Z* only. The imputation model, including predictors *X* and *Z* is expected to provide more sensible imputations.

## 5 Simulation results

In this section, we present the simulation results of the proposed diagnostic method under three different scenarios. First, we construct the nominal confidence interval for each observed data point based on the empirical distribution generated by the corresponding multiple imputed values. Then, for numerical assessment, we estimated the rate of coverage by which the nominal confidence intervals covers the observed data points (COV), the mean of the distance between the observed data and the mean of corresponding predictive posterior distributions (Distance), and the average width of the confidence intervals (CIW). The three metrics are the aggregated measures of individual posterior distributions. These metrics provide valuable information on the accuracy (distance), precision (CIW), and reliability (COV) of the diagnostic method to evaluate the adequacy of the imputation model. Using these metrics, researchers can determine if the diagnostic method provides valid and valuable information for making statistical inferences. Since the incomplete variable *Y* in section 4.1 is the conditionally normal distribution and the incomplete variable *X* in section 4.2 is the normal distribution, the mean of corresponding predictive posterior distributions is a valid representation of the center of corresponding posterior distributions. In such a case, the selected quantities (COV, Distance and CIW) could be used to inspect the discrepancy between observed and replicated data. We expect the better-fitted imputation model to derive a smaller Distance and CIW.

We also provided graphical analyzes with scatterplots, density plots, and distribution plots, which show observed values, upper and lower bounds of confidence intervals for each observed data point. The proposed diagnostic approach is performed on a variable-by-variable basis. Sometimes a single plot or summarized statistic is inadequate to arrive at a conclusion. Conducting PPC with various tools would provide a more comprehensive evaluation of the imputation model.

### 5.1 Quadratic equation with an incomplete outcome

Table 1 shows the results of the simulation study when the substantive model is a quadratic equation with an incomplete outcome. Since we only generated one incomplete dataset and repeated imputing it 50 times, all coverage rates were close to the pre-specified nominal level. However, when the imputation model was mis-specified as a linear regression model, the average distance was larger than the average distance under the correct specification of the imputation model (linear regression with a quadratic term). It conforms to our intuitive idea that the data would be close to the center of predictive posterior distributions if the model fits. The variance of the incomplete variable *Y* was set as 1, which implied that the width of 95% nominal confidence interval is approximately 3.92 (1.96 × 2) and the width of 75% nominal confidence interval is approximately 2.3 (1.15 × 2). When the imputation model was correctly specified, the estimated average width of the confidence interval was unbiased. However, the variance of *Y* was overestimated when the imputation model was linear.

**Table 1:** The rate of coverage by which the nominal confidence intervals cover the observed data points (COV), the mean of the distance between the observed data and the location of the predictive posterior distribution (Distance), and the average width of the nominal (75% and 95%) confidence intervals (CIW) for two imputation models (linear model and quadratic model) under different combinations of experimental factors. The analysis model is a quadratic equation with an incomplete outcome.

		COV				Average Distance				Average CIW			
		linear model		quadratic model		linear model		quadratic model		linear model		quadratic model	
	missingness	75%CI	95%CI	75%CI	95%CI	75%CI	95%CI	75%CI	95%CI	75%CI	95%CI	75%CI	95%CI
MCAR	30	0.76	0.96	0.75	0.93	2.41	2.41	0.79	0.79	6.64	11.32	2.27	3.87
	50	0.72	0.96	0.76	0.94	2.44	2.44	0.77	0.77	6.61	11.27	2.25	3.83
	80	0.77	0.95	0.78	0.94	2.25	2.25	0.87	0.87	6.53	11.13	2.51	4.28

MARr	30	0.75	0.95	0.74	0.94	2.28	2.28	0.8	0.8	6.26	10.66	2.31	3.94
	50	0.76	0.95	0.73	0.95	2.21	2.21	0.81	0.81	6.3	10.73	2.3	3.91
	80	0.8	0.96	0.77	0.92	1.8	1.8	0.83	0.83	5.43	9.25	2.38	4.05

**Figure 3:**
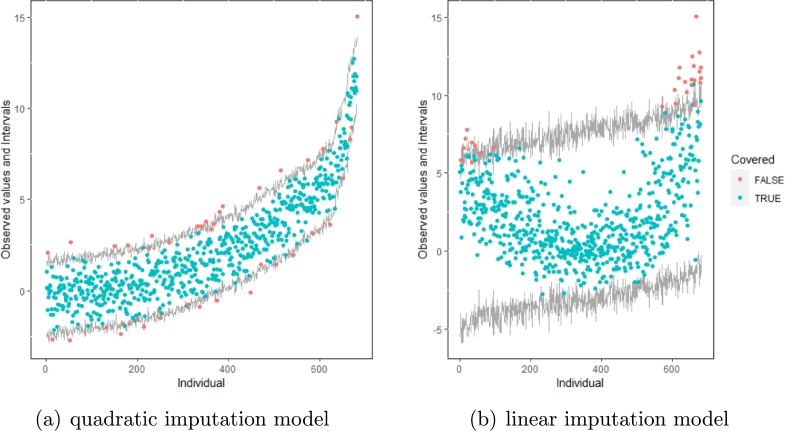
Distribution plots for the first simulation study (quadratic equation with an incomplete outcome) generated under 30% missing cases and MARr missingness mechanism. The confidence interval is 95% nominal. This plot provides upper and lower bounds (grey lines) of the posterior predictive distribution for all observed *Y* in ascending order of the expectation of the posterior distribution. Blue points imply the corresponding observed value falls in the interval, while red points imply the corresponding observed value falls outside the interval.

The same result could also be derived from the graphical analysis. Figs. 3(a) and 3(b) show distribution plots under the scenario of 30% missing cases and MARr missing mechanism. This plot provides upper and lower bounds of the posterior predictive distribution for all observed *Y* in ascending order of the mean of the posterior distribution. Blue points imply the corresponding observed value falls in the interval, while red points imply the corresponding observed value falls outside the interval.

**Figure 4:**
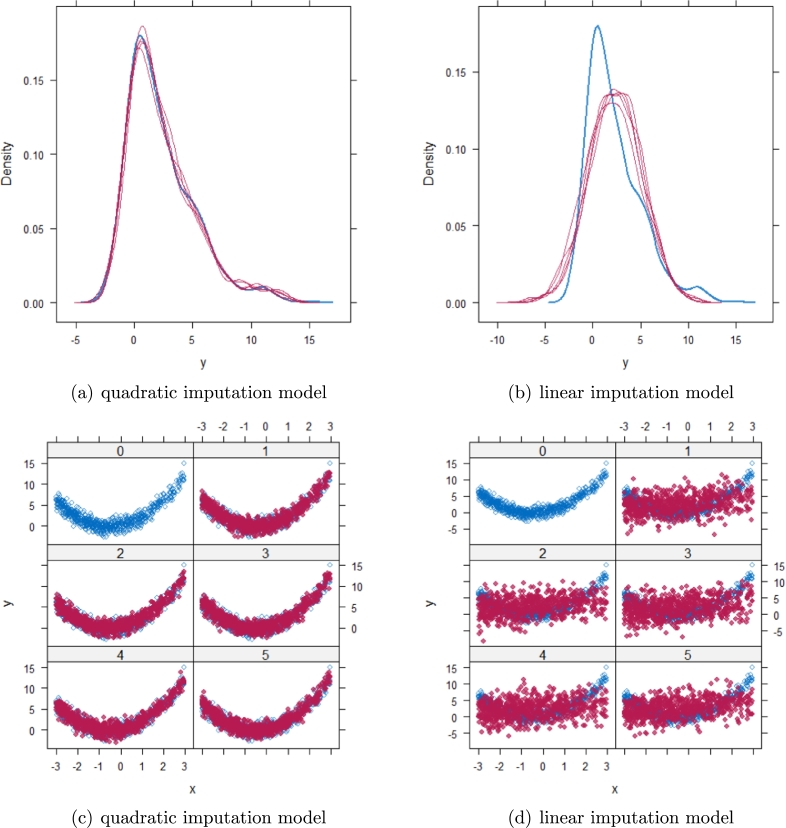
Scatterplots and densityplots for the first simulation study (quadratic equation with an incomplete outcome) generated under 30% missing cases and MARr missingness mechanism. Densityplots (a) and (b) show kernel density estimates for the distribution of the variable *Y* (blue) and *m* = 5 densities calculated from the imputed data (red). Scatterplots (c) and (d) show observed values (blue) of *Y* (label 0) and *m* = 5 comparisons of observed (blue) and imputed (red) values (label 1-5).

When the imputation model was correctly specified, the red points were randomly spread over the sample space without any patterns. However, when the imputation model was incorrectly specified as the linear regression model, the red points are shown at tails with lower and higher expectations of the posterior distribution. Moreover, the width of the intervals was generally narrower when the model was correct. The density plot and the scatter plot of the observed and replicated data generated with the function **densityplot()** and **xyplot()** in MICE also show the evidence that the quadratic regression is preferable to the linear model (see Figs. 4(a) to 4(d)). The scatter plot of the quadratic regression imputation model shows that replicated data overlapped the observed data. The density plot shows that the replicated data shared the same distribution as the observed data. This evidence illustrates the congeniality of the quadratic regression imputation model. However, the linear regression model performed worse than the quadratic regression model. First, the replicated data did not cover the observed data in two extreme regions in the scatter plot. Second, the empirical density of the replicated and observed data differed.

**Figure 5:**
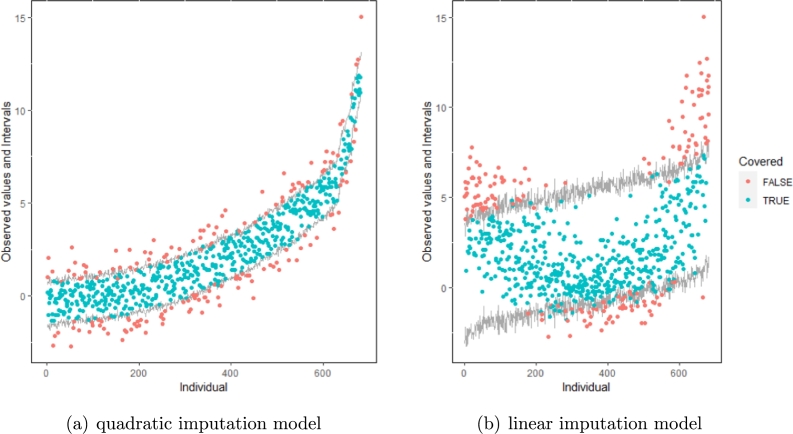
Distribution plots for the first simulation study (quadratic equation with an incomplete outcome) generated under 30% missing cases and MARr missingness mechanism. The confidence interval is 75% nominal. The confidence interval is 95% nominal. This plot provides upper and lower bounds (grey lines) of the posterior predictive distribution for all observed *Y* in ascending order of the expectation of the posterior distribution. Blue points imply the corresponding observed value falls in the interval, while red points imply the corresponding observed value falls outside the interval.

Furthermore, our proposed PPC approach for imputation models is robust against the different percentages of missing cases, missingness mechanisms, and the confidence interval's nominal levels. The nominal level of the confidence interval is determined by the extent to which we could undertake the outliers when the imputation model is not congenial with the data-generating process. For instance, there were more outliers in the plot of means and 75% confidence intervals than in the plot of mean and 95% confidence intervals. (See Figs. 5(a) and 5(b)).

### 5.2 Quadratic equation with incomplete covariates

Table 2, Table 3 show the result of the simulation for the quadratic equation with incomplete covariates. Based on the numerical results, the performance of these three methods, PC, SMC-FCS, and PMM, was the same, despite the slightly reduced coverage rate of PMM. In fact, when the missingness mechanism is MCAR (to bypass the problem of the sparse observed region for PMM), PMM would also provide a valid inference of the regression parameters (see Table 4) [25].

**Table 2:** The rate of coverage by which the nominal confidence intervals cover the observed data points (COV), the mean of the distance between the observed data and the location of the predictive posterior distribution (Distance), and the average width of the nominal 95% confidence intervals (CIW) for PC, SMC-FCS and PMM under different combinations of experimental factors. The analysis model is a quadratic equation with incomplete covariates.

		COV			Average distance			Average CIW		
	missingness	PC	SMC-FCS	PMM	PC	SMC-FCS	PMM	PC	SMC-FCS	PMM
MCAR	30	0.94	0.94	0.91	0.62	0.62	0.68	3.02	3.1	3.03
	50	0.94	0.93	0.9	0.61	0.61	0.67	3.03	3.06	2.97
	80	0.96	0.94	0.91	0.61	0.64	0.65	3.06	3.14	3

MARr	30	0.94	0.94	0.89	0.59	0.59	0.64	2.93	2.84	2.84
	50	0.93	0.94	0.89	0.57	0.58	0.62	2.74	2.7	2.68
	80	0.93	0.97	0.93	0.56	0.56	0.61	2.61	2.64	2.69

**Table 3:** The rate of coverage by which the nominal confidence intervals cover the observed data points (COV), the mean of the distance between the observed data and the location of the predictive posterior distribution (Distance), and the average width of the nominal 75% confidence intervals (CIW) for PC, SMC-FCS and PMM under different combinations of experimental factors. The analysis model is a quadratic equation with incomplete covariates.

		COV			Average distance			Average CIW		
	missingness	PC	SMC-FCS	PMM	PC	SMC-FCS	PMM	PC	SMC-FCS	PMM
MCAR	30	0.76	0.75	0.7	0.62	0.62	0.68	1.77	1.82	1.78
	50	0.75	0.75	0.73	0.61	0.61	0.67	1.78	1.8	1.74
	80	0.78	0.76	0.75	0.61	0.64	0.65	1.8	1.84	1.76

MARr	30	0.76	0.74	0.71	0.59	0.59	0.64	1.72	1.66	1.67
	50	0.74	0.72	0.69	0.57	0.58	0.62	1.61	1.58	1.57
	80	0.75	0.73	0.7	0.56	0.56	0.61	1.53	1.55	1.58

**Table 4:** The PMM performs under the scientific model : *Y* = *α* + *Xβ*_1_ + *X*^2^*β*_2_ + *ϵ*, where *α* = 0, *β*_1_ = 1 and *β*_2_ = 1. The error term and variable *X* follow standard normal distributions. 30% cases of *X* and *X*^2^ are designed to be jointly missing. The missingness mechanism is MCAR.

	True value	Estimates value	Coverage rate
*β*_1_	1	1.008	0.934
*β*_2_	1	1	0.958

**Figure 6:**
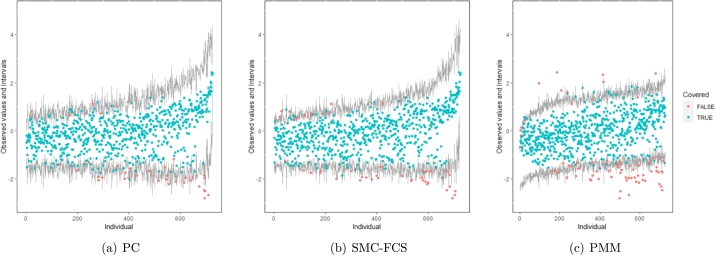
Distribution plots for the second simulation study (quadratic equation with incomplete covariates) generated under 30% missing cases and MARr missingness mechanism. The nominal level is 95%. The confidence interval is 95% nominal. This plot provides upper and lower bounds (grey lines) of the posterior predictive distribution for all observed *X* in ascending order of the expectation of the posterior distribution. Blue points imply the corresponding observed value falls in the interval, while red points imply the corresponding observed value falls outside the interval.

**Figure 7:**
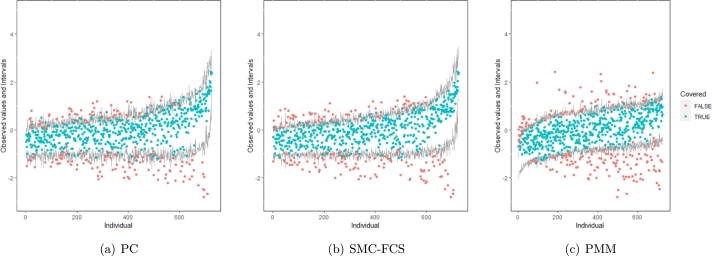
Distribution plots for the second simulation study (quadratic equation with incomplete covariates) generated under 30% missing cases and MARr missingness mechanism. The nominal level is 75%. This plot provides upper and lower bounds (grey lines) of the posterior predictive distribution for all observed *X* in ascending order of the expectation of the posterior distribution. Blue points imply the corresponding observed value falls in the interval, while red points imply the corresponding observed value falls outside the interval.

**Figure 8:**
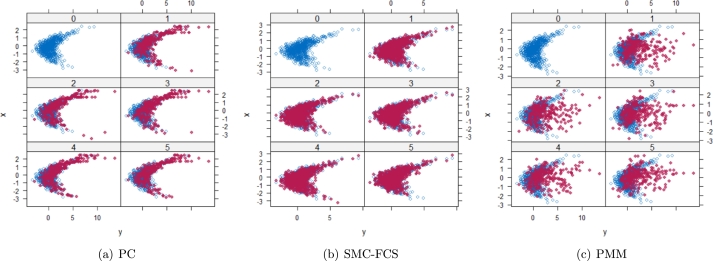
Scatterplots for the second simulation study (quadratic equation with incomplete covariates) generated under 30% missing cases and MARr missingness mechanism. Densityplots (a) and (b) show kernel density estimates for the distribution of the variable *X* (blue) and *m* = 5 densities calculated from the imputed data (red). Scatterplots (c) and (d) show observed values (blue) of *X* (label 0) and *m* = 5 comparisons of observed (blue) and imputed (red) values (label 1-5).

However, when it comes to graphical diagnostics, the misfit of PMM appears. The distribution plots (Figs. 6(a), 6(b), 7(a), and 7(b)) show that PC and SMC-FCS generated the same posterior predictive distribution of the observed data. There were more outliers with a larger value of *Y*. It is sensible since the density function of *X* based on *Y* is not monotone. Thus, it is unavoidable to impute the missing cell on the opposite arm of the parabolic function. Although the imputed value was not the same as the actual value in such a case, the replicated data still overlapped the observed data in the scatter plots (see Figs. 8(a) and 8(b)). The distribution plot of PMM with a 95% nominal level in Fig. 6(c) did not show more outliers than these of PC and SMF-FCS. However, when the nominal level was set to 75%, more outliers appeared in the sub-plot of PMM (Fig. 7(c)). The reason is that there are more observed data close to the center in the plots of PC and SMC-FCS, which implies the superiority of PC and SMC-FCS. The scatter plot also shows the discrepancy between the replicated and the observed data distribution for PMM (Fig. 8(c)). The result is robust against various percentages of missing cases and over the studied missing mechanisms.

### 5.3 Generalized linear model for discrete variables

**Figure 9:**
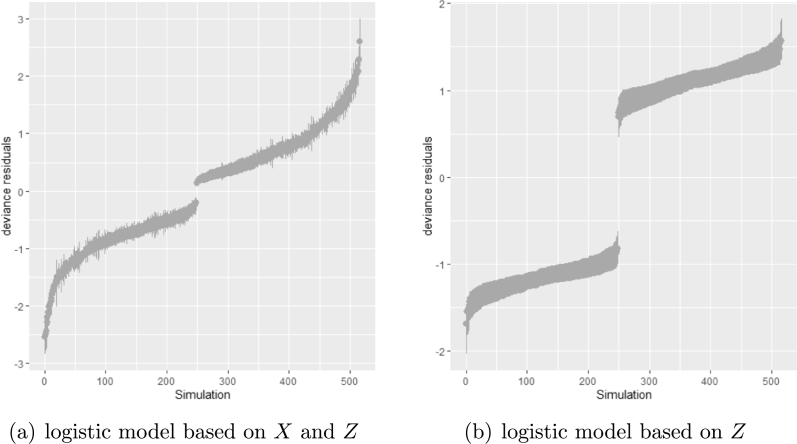
The plot of deviance residuals for the third simulation study (generalized linear model for discrete variables) generated under two logistic regression imputation models. The percentage of missing is 30%, and the missingness mechanism is MARr.

Table 5 shows the average sum of squared deviance for two logistic regression models. The value of the average sum of squared deviance was smaller when the imputation model was correctly specified with logistic regression on both *X* and *Z*. The result is robust against the percentage of missing cases and missingness mechanisms. Figs. 9(a) and 9(b) show that the residuals tend to zero when the imputation model fits the observed data better. The distribution of the observed data was more extreme than the empirical posterior distributions of replicated data generated under the logistic model with only variable *Z*.

**Table 5:** The average sum of squared deviance for two imputation models: 1) logistic regression with two predictors *x* and *z* 2) logistic regression with one predictor *x* under different combinations of experimental factors. The outcome is a dichotomous variable *y* and the binary regression is based on *x* and *z*.

		mean of residual deviance	
	missingness	with x	without x
MCAR	30	0.83	1.25
	50	0.85	1.27
	80	0.95	1.3

MARr	30	0.9	1.34
	50	0.94	1.35
	80	0.98	1.28

## 6 Application

### 6.1 Background

We illustrate the application of the proposed PPC for multiple incomplete variables with the data from the body mass index (BMI) of Dutch adults. This application is to study whether the proposed PPC works for a sequence of imputation models. More specifically, we aim to investigate whether the incorrect imputation model for one incomplete variable would disturb the proposed PPC for other variables. BMI is defined as the body weight divided by the square of the body height, broadly applied to categorize a person into underweight, normal, overweight, and obese. Since measuring a person's weight and height is costly, an alternative is to ask people to report their weight and height. However, such self-report data is systematically biased. People are used to overestimating their height and underestimating their weight, leading to a lower self-report BMI than measured BMI (van Buuren, 2018, section 9.3). The study aims to estimate unbiased BMI from the self-report data. We apply the multiple imputation approach to fill the unobserved measured weight and height.

The data we analyze is named selfreport in MICE package. The data consists of two components. One is the calibration dataset that contains data on 1257 Dutch individuals with both self-report and measured height and weight, which was taken from Krul, Daanen, and Choi [28]. The original survey included 4459 adults from either Italy, Netherlands, or North America aged 18-65 years in 1999 or 2000. The second part is a survey dataset that includes data from 803 Dutch adults aged 18-75 years with only self-reported data. The survey data were collected in November 2007, either online or using paper-and-pencil methods [29]. Six variables are included in the application: age (years), sex (male or female), hm denoting measured height (cm), hr denoting self-reported height (cm), wm denoting measured weight (kg), and wr denoting self-reported weight (kg).

To fit the aim of this application study, we design two linear regression imputation models for hm: one includes all the other variables, and the other includes all the other variables except the variable hr. Similarly, there are two linear regression imputation models for wm: one includes all the other variables, and the other includes all the other variables except the variable wr. In such a case, we have four imputation strategies to evaluate:

1. Case 1: include hr in the imputation model of hm and wr in the imputation model of wm.
2. Case 2: include hr in the imputation model of hm and exclude wr from the imputation model of wm.
3. Case 3: exclude hr from the imputation model of hm and include wr in the imputation model of wm.
4. Case 4: exclude hr from the imputation model of hm and wr from the imputation model of wm.

Although it is evident that hr and wr are significant covariates for the imputation of hm and wm, we deliberately ignore these two variables in some cases to evaluate whether incorrect imputation model for hm (wm) influences PPC for wm (hm). If the target of analysis is BMI, one could apply passive imputation to include BMI in the imputation process (van Buuren 2018, section 6.4). In such a case, BMI is still not considered as the predictor of hm and hm because of linear dependencies.

### 6.2 Results

**Figure 10:**
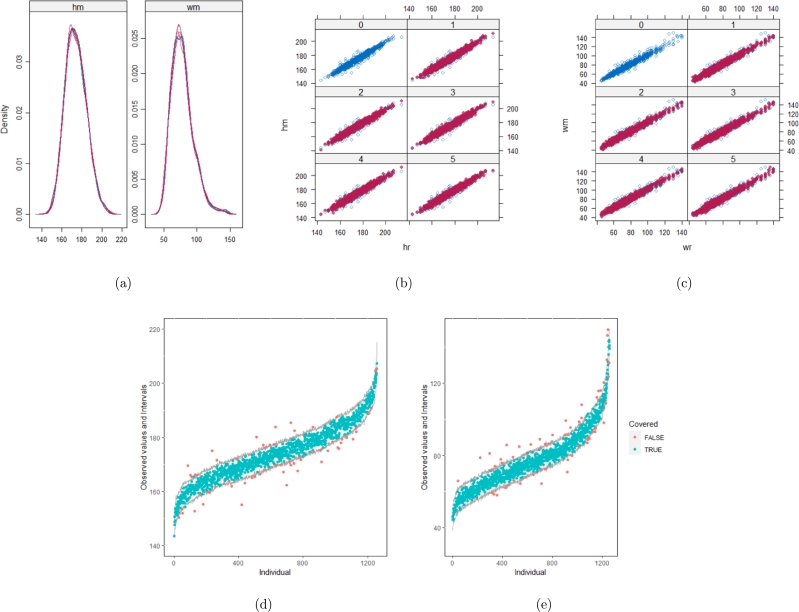
Graphical analysis of the BMI data with imputation strategy case 1. (a) density plots, (b) scatter plot of hm, (c) scatter plot of wm, (d) distribution plot of hm, and (e) distribution plot of wm.

**Figure 11:**
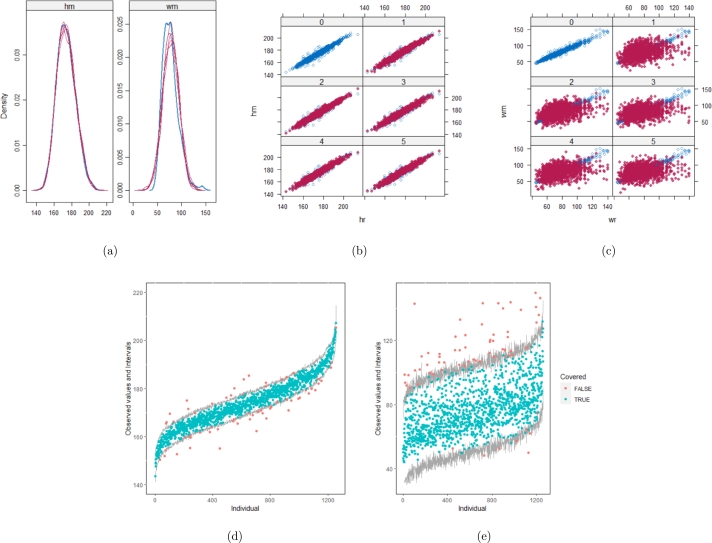
Graphical analysis of the BMI data with imputation strategy case 2. (a) density plots, (b) scatter plot of hm, (c) scatter plot of wm, (d) distribution plot of hm, and (e) distribution plot of wm.

**Figure 12:**
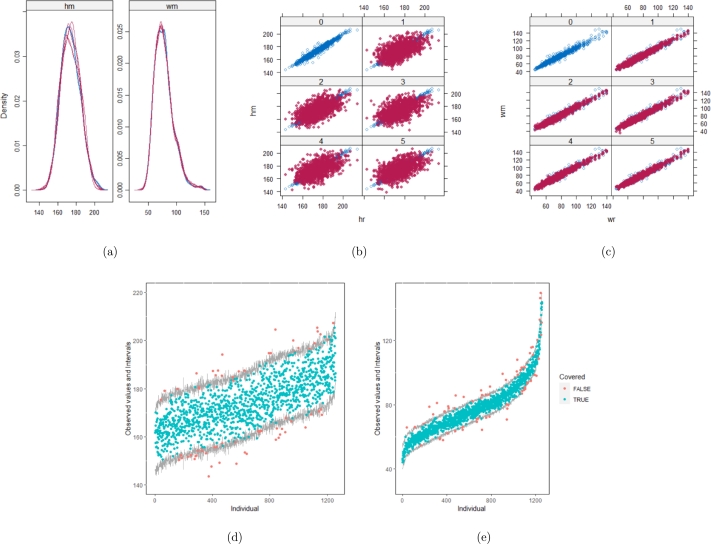
Graphical analysis of the BMI data with imputation strategy case 3. (a) density plots, (b) scatter plot of hm, (c) scatter plot of wm, (d) distribution plot of hm, and (e) distribution plot of wm.

**Figure 13:**
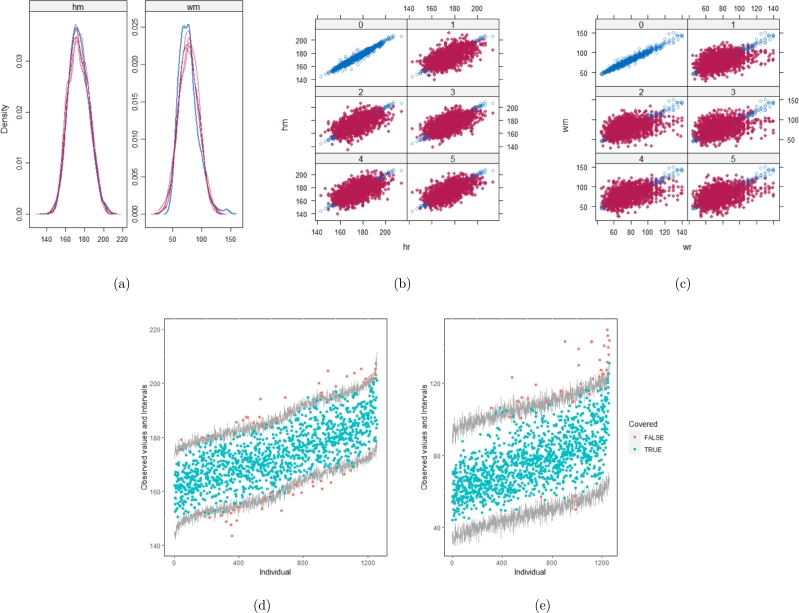
Graphical analysis of the BMI data with imputation strategy case 4. (a) density plots, (b) scatter plot of hm, (c) scatter plot of wm, (d) distribution plot of hm, and (e) distribution plot of wm.

Table 6 shows that the best imputation model among these four is the one that includes both wr and hr. The average distance and the width of confidence intervals for the observed data were the smallest for both hm and wm. No matter whether the imputation model of hm was correctly specified, the linear regression imputation model for wm should be based on all the other variables. When fixing the imputation model for the hm (no matter whether including hr or not), the average distance and the average width of the confidence interval of hm derived under the linear model included hr was remarkably less than the result taken under the linear model excluded the covariate hr. The graphical results (Figs. 10(a) to 10(e), 11(a) to 11(e), 12(a) to 12(e), and 13(a) to 13(e)) show the same conclusion. When the linear regression imputation model for wm or hm was correctly specified, the imputed data overlapped the observed data in the scatter plot. The observed data would be closer to the center of the confidence interval, and the width of the confidence intervals was relatively small. However, the result of wr in case 3 was slightly larger than in case 1. Similarly, the result of wr in case 4 was slightly larger than in case 2. A similar result could be found in fixing the imputation model for the wm (no matter whether the imputation model includes wr or not). The average distance and the average confidence interval of wm derived under the linear model had wr were remarkably less than the result taken under the linear model excluded the covariate wr.

**Table 6:** The performance of 4 imputation strategies is summarized by the coverage rate, the average distance and the average width of confidence intervals with respect to missing variables hm and wm. hm denotes measured height (cm). wm denotes measured weight (kg). We estimated the rate of coverage by which the nominal confidence intervals covers the observed data points (COV), the mean of the distance between the observed data and the mean of corresponding predictive posterior distributions (Distance), and the average width of the confidence intervals (CIW).

	hm			wm		
	cov	average distance	average CIW	cov	average distance	average CIW
strategy 1	0.95	1.57	8.27	0.95	2.28	12.46
strategy 2	0.95	1.65	8.89	0.94	10.9	54.38
strategy 3	0.95	5.58	26.89	0.94	2.35	12.83
strategy 4	0.95	5.56	27.88	0.97	9.84	59.57

The findings imply that incorrect specification of the imputation models for other incomplete variables $Y_{-j}$ would influence the target variable $Y_{j}$ for which we perform the PPC because densities of the imputed variables $Y_{-j}$ are different from the ‘true’ densities. However, we can still select the correct model for $Y_{j}$. Our application scenario is relatively simple: the linear model is sufficient to reflect the data-generating process of incomplete variables. However, we do not rule out the possibility that under extreme and complicated cases, incorrect specification of the imputation models for other incomplete variables $Y_{-j}$ would prevent us from selecting the most suitable imputation model for the incomplete variable $Y_{j}$.

## 7 Discussion

The proposed imputation model diagnostic procedure based on PPC involves numerical assessment and graphical analysis. Overcoming the limitations of the method proposed by He & Zaslavsky [8] and Nguyen et al. [9], this approach is suitable for fully conditional specification and insensitive to the degree of missingness. Notably, applying both numerical and graphical tools benefits a thorough understanding of model selection. For numerical assessment, the evidence of a fitted imputation model is less deviation between the observed value and the expectation of corresponding predictive posterior distribution and narrower width of confidence intervals of predictive posterior distributions for the observed data. For graphical analysis, we provide the distribution, scatter, and density plots. The more suitable imputation models are, the more similar the replicated data to the observed data in the density and scatter plots. The distribution plot shows the posterior distributions of all observed data. It allows the researcher to identify the regions where the imputation model misfits. Furthermore, the graphical analysis could be applied to evaluate whether a specific imputation model is adequate to provide plausible imputations.

The simulation study demonstrates that the proposed imputation model diagnostic procedure works on continuous and discrete variables under parametric and hot-deck multiple imputation approaches. For continuous variables, the distribution plots can be used to derive information to improve the imputation model. For example, although the imputation model is incorrect, it would provide valid imputations in the focused regions. In such a case, we could still apply the suboptimal imputation model. Moreover, we could also adjust the imputation model in the misfitted regions and develop a piecewise imputation model. However, the PPC for categorical data or ordered categorical data is limited since the predictor of the imputation model is the probability of assignment rather than the observed data itself. Therefore, we currently investigate residual deviance as the indicator to select the model for categorical data and ordered categorical data.

For hot-deck imputation approaches, what PPC diagnoses do are the donor-selection procedure. As a result, shown in section 5.2, selecting donors for the composition $X+X^{2}$ performed better than only solving for the incomplete variable *X*. SMC-FCS was treated as the baseline in our simulation since it is proven as a reliable imputation method when the substantive model is known [26]. The PC performs as well as the SMC-FCS, which implies the donor selection process of PC reflects the data generating process in our simulation scenarios. However, based on the features of predictive mean matching, the appropriate donor selection does not ensure plausible imputations. Extra analysis of the observed and imputed data would then be necessary.

The application example shows that the PPC works on incomplete multivariate datasets. When the imputed covariate deviates from the actual distribution because of the mis-specified imputation models, the imputation model for the predictor could also be selected by PPC. In our case study, the mis-specification of one incomplete variable slightly influences the other incomplete variable's numerical results. However, in more extreme situations, such as a large number of incomplete variables and more ridiculous imputation models for covariates, the result may be influenced seriously to result in a sub-optimal model selection. Therefore, it is more reasonable to perform the numerical analysis of all incomplete variables and make the model selection for those variables with remarkably different results under different candidate imputation approaches first.

Existing PPC proposed by He & Zaslavsky [8] and Gelman et al. [7] measured the posterior predictive p-value to indicate the discrepancies of summarized statistics between the observed and replicated data. The close to 0 or 1 p-value implies the inadequacy of the imputation model for the target quantities. The target quantities should be calculated with the completed data, which consists of the observed and the imputed data, because it allows the researcher to calculate the target quantities requiring a complete data matrix. Both He & Zaslavsky [8] and Nguyen et al. [9] found that the existing PPC for multiple imputation models is sensitive to the percentage of missing cases. Since the imputed and replicated data are generated from the same posterior predictive distribution, evaluation becomes more difficult with an increasing proportion of missing data.

Unlike the existing PPC approach, the PPC discussed in the paper checks the imputation model for each incomplete variable under the FCS framework. In addition, we diagnose the distribution of the observed data so that the result would also be reliable with a large proportion of missing cases. The simulation study also shows that the proposed PPC works for different missingness mechanisms. However, since posterior predictive checking can only evaluate the observed data, it cannot be used when the missingness mechanism is missing not at random (MNAR, [30]). Also, additional diagnostics are required for imputation methods that can not extrapolate, such as a distribution comparison between observed and imputed data.

The PPC for multiple imputation models based on target analysis would be more informative for “one-goal” studies. The imputer is also the analyst with a specific scientific interest in such a case. The diagnosis procedure aims to select imputation models to produce imputations that could support the particular post-imputation analyzes. However, the diagnostic method proposed in this paper is designed for “multiple goals” studies. The imputer may not know the potential research on the imputed data. Our approach aims to select congenial imputation models to ensure that Rubin's rule will provide a valid inference. The imputed data generated by selected imputation models could be used for more general downstream analysis and different scientific interests.

When the sample size is tremendous, it is better to choose some representative data to check the imputation model so that the scatter plot or the distribution plot would not be too crowded. A clustered procedure could be applied to gather the observed data with closed values and choose one subset in each cluster to check the model. Further investigation is necessary to set the rule to select the observed data when the sample size is too large.

## Funding

No funding source has been utilized in this work.

## CRediT authorship contribution statement

All authors listed have significantly contributed to the development and the writing of this article.

## Declaration of Competing Interest

The authors declare that there is no conflict of interest.

## Data Availability

The data used in the article is simulation data. The details are available from the GitHub repository: https://github.com/Mingyang-Cai/PPC.

## Appendix A Supplementary material

MMC The data used in the article is simulation data. The details are available from the GitHub repository: https://github.com/Mingyang-Cai/PPC. MMC
