# Supplementary material for: Graphical and numerical diagnostic tools to assess multiple imputation models by posterior predictive checking
Source: Heliyon. 2023 Jun 13;9(6):e17077. doi: 10.1016/j.heliyon.2023.e17077 (PMC10285146; doi:10.1016/j.heliyon.2023.e17077)
Supplement: MMC — The data used in the article is simulation data. The details are available from the GitHub repository: https://github.com/Mingyang-Cai/PPC. [file mmc1.docx]

The data used in the article is simulation data. The details are available from the GitHub repository: https://github.com/Mingyang-Cai/PPC.
